# Advancing long-term phytoplankton biodiversity assessment in the North Sea using an imaging approach

**DOI:** 10.1038/s41597-025-06278-w

**Published:** 2025-12-16

**Authors:** Rune Lagaisse, Nick Dillen, Dias Bakeev, Wout Decrop, Paul Focke, Jonas Mortelmans, Julie Muyle, Klaas Deneudt

**Affiliations:** 1 Flanders Marine Institute (VLIZ), Jacobsenstraat 1, 8400 Oostende, Belgium; 2 Ghent University, Department of Biology, Laboratory of Protistology & Aquatic Ecology, Ghent, Belgium

**Keywords:** Marine biology, Biodiversity

## Abstract

This paper presents a high spatial and temporal resolution microphytoplankton long-term biodiversity assessment for the southern bight of the North Sea obtained by FlowCam imaging. We describe the extension of the time series with the release of over six years of new quality-controlled data as well as a taxonomic revision of previously published data leading to 92 newly recognized groups. We also describe the latest fine-tuning of sampling and laboratory processing protocols leading to a more robust methodological framework while maintaining time series continuity. The implementation of semi-automated data pipelines, leveraging convolutional neural networks, allows to deal with the high influx of biodiversity imaging data and metadata. Data and provenance metadata are annually published under a CC-BY license in trusted repositories. This current open access, high-resolution 7 year-long dataset serves as a valuable tool for studying phytoplankton communities in the Belgian Part of the North Sea.

## Background & Summary/Introduction

Phytoplankton are vital to marine systems and play a key role in the Earth’s biogeochemical cycles and climate. They contribute to 45% of global net primary production and their pivotal role in food webs underscores their significance. Phytoplankton respond quickly to environmental changes due to their high abundances and short generation times. Changes in abundance and composition of phytoplankton communities are transferred through trophic levels, impacting whole ecosystem functioning^1–3^. As environmental conditions change, phytoplankton have the potential to invade and disrupt ecosystems including increased occurrences of Harmful Algal Blooms (HABs). These HAB events can lead to mass mortality of marine life by release of toxins, nuisance effects or by hypoxic and anoxic conditions during decomposition of the bloom by bacteria^4–6^. Hence, monitoring phytoplankton is essential for assessing marine ecosystem health and safeguarding socio-economic assets. Regulatory frameworks like the Marine Strategy Framework Directive (MSFD) and the Water Framework Directive (WFD)^7,8^ use phytoplankton as a key indicator for assessing water quality, food webs and pelagic habitats^9–11^. However, the successful implementation of these indicators depends on the availability of long-term, high taxonomic resolution phytoplankton datasets^12^.

**Fig. 1:**
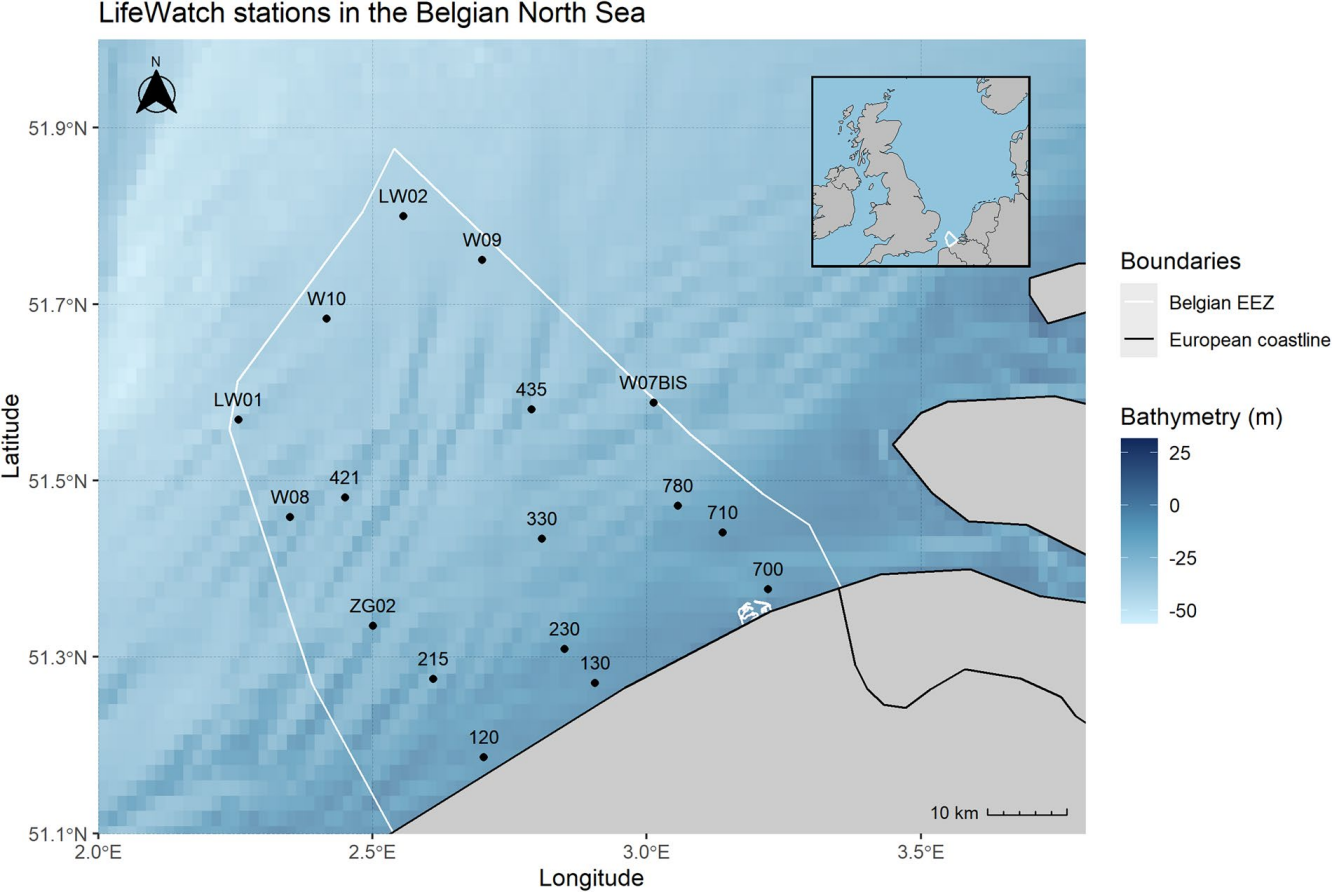
Sampling sites in the Belgian part of the North Sea (BPNS). Top-right insert indicates location of the study area in the North Sea. The colour bar represents the bathymetry in meters. The nine nearshore sampling stations 120, 215, ZG02, 130, 230, 330, 700, 710 and 780 are visited monthly. The eight offshore sampling stations W08, 421, LW01, 435, W10, W07BIS, W09 and LW02 are visited seasonally. The white polygon delineates the Belgian exclusive economic zone.

Phytoplankton are a morphologically and taxonomically diverse group, encompassing both photosynthetic protists and cyanobacteria. Within the Eukaryote domain phytoplankton span the SAR (Stramenopila, Alveolata, Rhizaria), Archaeplastida, Haptophyta and Cryptophyceae^13^. For the Belgian Part of the North Sea (BPNS), the dominant phytoplankton groups reported in terms of biomass are diatoms, dinoflagellates and prymnesiophytes^14–16^. The BPNS phytoplankton communities are characterized by a seasonal succession of blooms, mainly determined by light availability, temperature, nutrient conditions and to lesser extent by water column mixing and grazing. Spatial variability in the phytoplankton community structure is shaped by water masses influx, depth and turbidity^14,15,17^. Phytoplankton community structure and bloom onset exhibit interannual variations, linked to changes in sea surface temperature, light availability, nutrient ratios, and abundance of grazers^18–20^. The BPNS is a heavily impacted marine region, and subjected to many anthropogenic pressures, including offshore industry and construction, pollution, eutrophication, climate change, shipping and fishing^21^. These pressures exert changes in marine ecosystems, cascading down to the level of marine producers^22–24^. Despite the importance of phytoplankton monitoring, its nature is often short term and project-based. Long-term datasets are sparse, have gaps or lack spatio-temporal and/or taxonomic resolution^25^. For the BPNS, phytoplankton community composition has been monitored at station 330 (Fig. 1 from 1988 to 2008^16,17,26–28^. Since 2002, monthly pigment samples have been collected and analysed under the European LifeWatch program using High Performance Liquid Chromatography (HPLC) for studying functional phytoplankton groups at a lower taxonomic resolution^14^. The 4DEMON project (https://www.4demon.be), running from 2014 to 2018, aimed to integrate and centralise marine data collected in the BPNS over the last four decades. Under this program, a phytoplankton community composition dataset named the ‘Belgian Phytoplankton Database’ was constructed. This database is a compilation of data from technical reports, monitoring programs, projects, theses and PhD studies dating back to 1968 which were submitted to various control stages, including removal of digitalisation errors and duplicates, outlier checks, matching against a taxonomic backbone and replenishing and standardizing metadata^19,20^. Although the above-mentioned studies offer valuable insights, they are limited by temporal and spatial constraints and/or the main focus is often on higher taxonomic levels or nuisance algae like *Phaeocystis*.

Since May 2017, phytoplankton has been sampled in the BPNS during the monthly LifeWatch multidisciplinary campaigns and analysed using FlowCam (FluidImaging Technologies) imaging. This automated high-throughput device integrates the principles of flow cytometry, microscopy and imaging^29^. FlowCam operates by aligning particles in a constant fluid stream using a dosage pump and tubing system and by segmenting multiple particles in the Field of View (FOV) of a camera pointed at a glass photo chamber yielding images with an average resolution of 7140 px. FlowCam, in combination with associated in-house developed dataflows including semi-automatic classifiers, facilitates rapid, semi-automated and reproducible particle analysis, saving much time and effort compared to traditional microscopic monitoring but at the cost of lower image resolution. Building upon initial protocols detailed in Amadei Martínez *et al*.^30^, this paper describes recent enhancements of sampling and laboratory protocols leading to a more robust methodological framework, alongside implementation of semi-automated data pipelines for processing large volumes of data. This paper presents the extension of the dataset produced by Amadei Martínez *et al*.^30^ with the release of six additional years of quality-controlled data, and it includes a taxonomic review of historical data. The current 7.5 year long biodiversity occurrence dataset is unique in the BPNS and Southern Bight of the North Sea in terms of temporal and spatial coverage.

## Methods

### Sampling

Since May 2017, phytoplankton samples have been collected during the multidisciplinary LifeWatch sampling campaigns aboard the Research Vessel (RV) Simon Stevin. Nine onshore stations are sampled on a monthly basis, and eight additional offshore stations are sampled seasonally (every three months) (Table 1, Fig. 1). During these campaigns, samples for phytoplankton^30,31^ and zooplankton^32^ are collected to assess biodiversity occurences and abundances. These samples are complemented with a suite of water quality related parameters including measurements of pigments, nutrients, turbidity^33^, DNA, eDNA, water column measurements from CTD-profiling and underway measurements as measured by the RV Simon Stevin.

**Table 1:** LifeWatch sampling stations and coordinates, onshore stations are visited on a monthly basis, offshore stations are visited on a seasonal basis.

Onshore stations	Longitude, Latitude	Offshore stations	Longitude, Latitude
130	2.90535, 51.27055	LW01	2.256, 51.568667
230	2.85035, 51.308683	LW02	2.556, 51.8
330	2.809083, 51.434117	435	2.790333,51.580667
700	3.221017, 51.377	W07BIS	3.012517,51.588033
710	3.138283, 51.441217	W08	2.35, 51.458333
780	3.057283, 51.471367	W09	2.7, 51.75
120	2.702483, 51.186083	W10	2.416667, 51.683333
215	2.61075, 51.274867	421	2.45, 51.4805
ZG02	2.500717, 51.33515		

On board the RV Simon Stevin, the Marine Information and Data Acquisition System (MIDAS) allows to register sample metadata, navigation data, and meteorological data. Scientists log their onboard actions into the MIDAS system, ensuring that sample types and measurements are documented with real-time coordinates and timestamps, and they are assigned unique and traceable identifiers.

During the campaigns, phytoplankton samples for FlowCam imaging are collected by hauling 70 L (from May 2017 to May 2018) or 50 L (from June 2018 to August 2024) of surface water using stainless steel buckets, and filtered with a 55 µm mesh size Apstein net (Hydro-Bios, 1.2 m long, 0.5 m diameter). The filtered sample is transferred from the cod-end of the net to a 1 L plastic container and fixed with acid Lugol’s iodine solution, to a 5% final concentration for sampling conducted between May 2017 and June 2021. From June 2021 onwards, samples are fixed to a 1% final concentration of acid Lugol iodine solution, as heavy staining makes identification of some taxa, like dinoflagellates, more cumbersome and sample laboratory processing is typically enforced within 6 months after sampling. Following collection onboard, samples are stored in dark conditions and refrigerated at 4 °C until laboratory processing. Choice of lab processing post campaign rather than fresh processing at sea is driven by the limited transit time between the stations which would result in a build-up of samples and the risk of damaging sensitive equipment, sample spillage from the pipette tip and blurry images during rough weather conditions at sea.

**Fig. 2:**
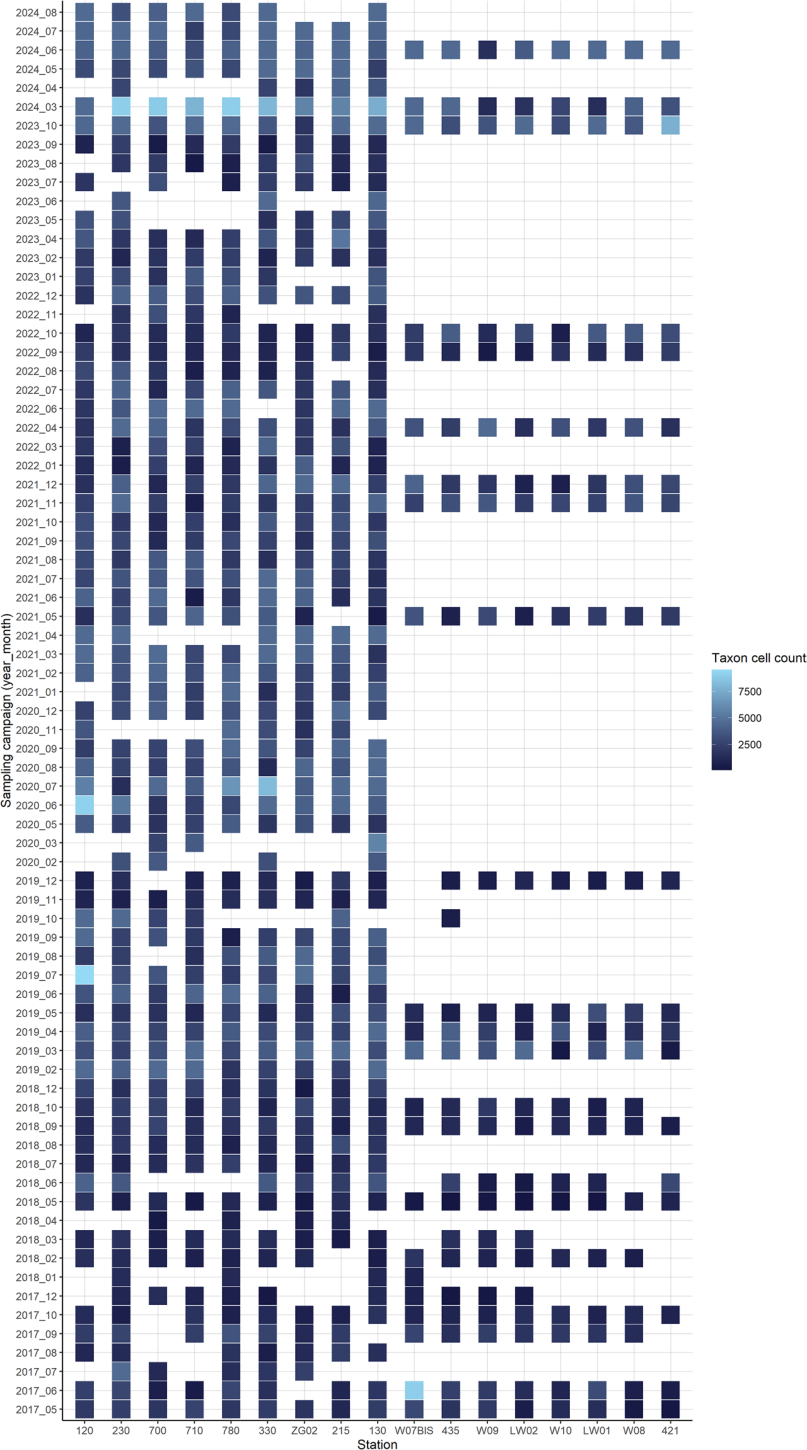
FlowCam phytoplankton biodiversity assessment data availability per station from May 2017 to August 2024. Color gradient indicates total cell count per sample over all taxa and gives an indication of cell sample load.

Spatio-temporal data availability is heterogeneous, with near-shore stations being visited at a monthly frequency and offshore stations at a seasonal frequency. Additional gaps in data availability are mainly due to harsh weather conditions in winter, Covid-19 restrictions and RV maintenance (Fig. 2).

### Lab processing

After collection, samples are processed in the onshore lab using the FlowCam VS-4 benchmodel (10219 serial number, Fluid Imaging Technologies, Yarmouth, Maine, U.S.A.) equipped with a Sony XCD SC90 digital gray-scale camera (1,280 × 960 camera resolution), C70 syringe pump model and VisualSpreadsheet® software version 4.2.52. To process the LifeWatch time series, the FlowCam is mounted with a 300 µm disposable flow cell, 4X magnification objective, 5 ml syringe and 5 ml pump. Each sample is prefiltered at 300 µm to avoid clogging of flow cells. We opted to process samples using the AutoImage mode, as opposed to autofluorescence mode, because of Lugol fixation. Context file settings are set to a frame rate of 20 frames per second and a flow rate of 1.7 ml/min, yielding an efficiency of about 41%, the highest possible efficiency for this FlowCam model using disposable 300 µm flow cells. These settings were selected because other combinations of flow- and frame rate exceed the manufacturer’s recommended efficiency, or significantly increase run time, from 8.82 to 30 minutes, to only increase efficiency by less than 1%. The basic acquisition filter is set to 70–300 µm Equivalent Spherical Diameter (ESD) from May to December 2017 and corrected to 55–300 µm ESD from January 2018 onward, with the upper size limit reflecting the width of the flow cells and the lower limit the Apstein net mesh sizes used at sea for sampling. Focusing on the sample is done manually, first by loading a sample in the field of view (FOV) of the camera and moving the flow cell holder on the rail. This is followed by performing fine focus via the’Setup and Focus’ menu, select ‘Tools’ and ‘Enable Manual Focus’. This enables the flow cell to be moved on the rail in steps of 10 µm for precise focusing. This manual focusing method allows us to focus on the main cell shape of each sample, as opposed to the spherical manufacturer beads used in the automated focussing procedure. We opt to only save image collages for downstream identification of particles. Raw images and binary collages are not saved due to the large amounts of storage required.

After manual focussing, particle load of the sample is determined during a presample run of 1.5 ml. Based on the particles per used image (PPUI) returned in the run summary, the sample is diluted to a PPUI between 1.1 - 1.2 to avoid particles overlapping in a single frame, as advised by the manufacturer (FluidImaging Technologies, FlowCam manual version 3.4, June 2014). Once diluted, three replicate sample runs are performed to balance technical variation. Each replicate run has a stop criterion of 1,500 captured particles or a sample volume processed of 5 ml from May to Dec 2017, 8 ml for the period Jan 2018 to May2021, and 15 ml from June 2021 onwards to allow us to capture more cells in low load samples while keeping run time just below 30 minutes^34,35^. During the sample runs, sporadic pinching of the tubes is performed to avoid clogging or to release particles that are visibly stuck to the flow cell wall. This current combination of device settings allows us to produce a complete image library of a sample in under 30 minutes run time. Between presample runs, replicate runs, and different samples, the flow cell and tubing system is alternately rinsed 3 times with 5 ml of ethanol 70% and 5 ml of Milli-Q water, leaving little air in between fluids to avoid mixing, and always ending with Milli-Q. After processing water samples are stored refrigerated and in dark conditions for biobanking for 2 years and are available upon request. The full sampling and laboratory protocols, including details on all context file settings, are open-access and published in protocols.io and available via 10.17504/protocols.io.6qpvr8e6zlmk/v1^36^.

### Data processing

**Fig. 3:**
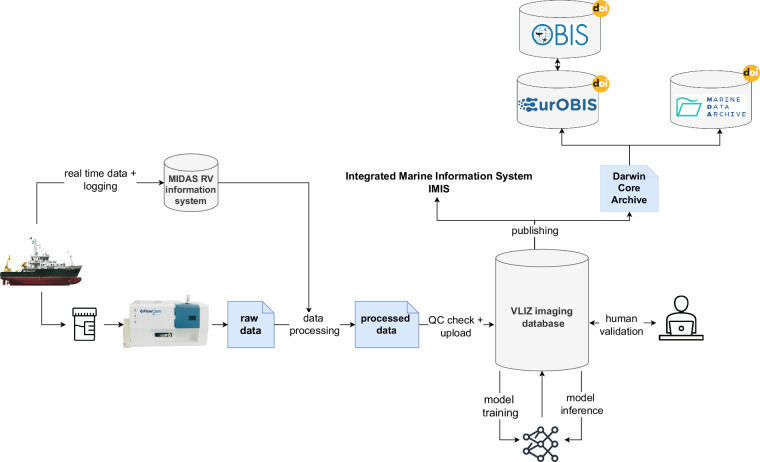
Schematic overview of FlowCam data acquisition, processing and publishing. During sample collection at sea, real time data and actions are logged in the RVs MIDAS information system. After sample processing in the lab, raw FlowCam data is processed to extract relevant data and metadata and linked to the real-time sample data. All processed data is quality controlled and uploaded to an internal processing database. Newly uploaded data is given to a pretrained model for inference, after which scientists manually check image labels. Human checked data is aggregated to taxon counts per sample and transformed to a DarwinCore Archive for publication in EurOBIS, OBIS^40^ and the Marine data Archive^41^ for further dissemination to end users. Dataset records are assigned a DOI and logged in the IMIS^42^ catalogue for discoverability.

To efficiently manage and process the annual influx of 300,000–400,000 particle images coming from the FlowCam biodiversity surveys in the BPNS, semi-automatic data pipelines were set-up and integrated with deep learning algorithms (Fig. 3). After laboratory processing, images and metadata are harvested from raw FlowCam output directories, checked and formatted using a set of Python scripts. This includes cropping of the raw image collages to isolate single Regions Of Interest (ROI’s) without removal of the background of the ROIs, retrieving metadata regarding laboratory processing from the raw run summary and context files, and image parameters from a raw list (.lst) file. Subsequently, the textual output from these file types is compiled, reformatted, and linked to the real-time sampling metadata pulled from the ships’ MIDAS vessel information system onboard RV Simon Stevin. All cropped images and formatted metadata is quality control checked before upload to an in-house MongoDB processing database. These checks include matching field names and value types against the predefined database scheme and checking for missing or illogical values. The BioSenseMongoDB database is an in-house NoSQL document database specifically set-up for processing and labelling images, it does not act as a data archive. The BioSenseMongoDB processing database hosts collections of individual FlowCam ROIs and a rich set of over 249 metadata fields associated with each image related to sampling, lab processing and classification. This extensive set of metadata allows for logical query options for scientists, data quality checks, and it supplies metadata input for analysis.

Using input data from the BioSenseMongoDB database, Convolutional Neural Networks (CNNs) are trained on human labelled image data and subsequently, new incoming image data can be classified using these pretrained models. Model training and inference processes are executed on a virtual TensorFlow server hosted at VLIZ, featuring 2x4GB GPU addresses and 32GB virtual memory. After inference, each resulting predicted image is manually checked and corrected where necessary by scientists using an in-house labelling before publication of data, all data published has been validated by humans after the initial model prediction step. The labelling tool allows scientists to easily query for predicted images and write corrected labels directly to the BioSens MongoDB database via a graphical user interface. Over the years, a CNN based on the off-the-shelf Xception architecture^37^ has been annually retrained on the increasing FlowCam human validated image library, currently comprising 2 million validated images to sample training data splits from, to increase performance. Latest model versions can recognize up to 95 FlowCam groups with a top-two accuracy exceeding 93%.

Once all images of a monthly survey are validated by scientists, the biodiversity occurence data is aggregated to yield taxon counts and taxon densities per sample. Cell densities per litre of seawater are calculated following Eq. 1 from Amadei Martínez *et al*.^31^:

$${Density}\left(\frac{{particles}}{L}\right)=\frac{{Particle\; Count}\ast {Sample\; Volume}({mL})}{{Fluid\; Volume\; Imaged}\left(L\right)\ast {dilution\; factor}\ast {Volume\; Filtered}(L)}$$

Equation 1: FlowCam density calculation, from Amadei Martínez *et al*.^31^.

where Volume Filtered is volume of seawater filtered through the Apstein net at sea, Sample Volume is the volume of the sample after filtration through the Apstein net measured in the lab, Fluid Volume Imaged is the total volume of sample that was captured in the field of view of the camera (i.e. sum of fluid volume captured per frame for a sample, measured by FlowCam), Particle Count is the number of pictures taken of each taxon in the sample and dilution factor is the dilution performed if the sample exceeded a PPUI of 1.2.

### Taxonomic structure and revision of the dataset

In Amadei Martínez *et al*.^30^, the taxonomic coverage of the FlowCam dataset for the years 2017 and 2018 comprised 55 groups, including 33 diatom groups, 7 dinoflagellate groups, and 15 non- phytoplankton groups. In recent years, manual revisions and updates have refined the taxonomic composition of the full dataset. These changes include i). splitting higher taxonomic level groups into lower level groups where image resolution was sufficient to distinguish genus or species level identification, ii). excluding lower level taxonomic groups for which deterministic morphological characteristics are insufficiently visible in FlowCam images, iii) additional higher-level taxonomic groups were introduced to ensure there are always higher-level labels to resort to when required morphological characteristics are invisible for genus or species level classifications in a specific image iv) certain labels were renamed to encompass two or three species or genera that cannot be distinguished in FlowCam due to inadequate resolution or because of their (semi-)cryptic nature. *Chaetoceros curvisetus* was renamed to *Chaetoceros curvisetus/pseudocurvisetus* for example. This new set of labelling groups were used during validation of FlowCam data from 2020 onwards. Previously validated data from 2017, 2018 and 2019 were subsequently revised accordingly to guarantee continuity in the time series. The fully revised dataset ranging from May 2017 to August 2024 now comprises 138 biological groups, including 76 diatom groups, 17 dinoflagellate groups, 16 ciliate groups, 9 other phytoplankton groups, 16 zooplankton groups and 4 other groups (Supplementary table 1). A number of non-biological particles is also recognized but not included in aggregated published datasets. ‘Artefact’, ‘Replicate’, ‘Aggregate’, ‘Bubbles’, ‘Remnant, ‘Replicate’ groups are omitted from aggregations because they are artefacts of the FlowCam run in the lab and don’t present any relevant biological information. A number of inorganic and organic particles are also classified rudimentary into ‘Detritus’, ‘Fibers’ and ‘Mineral particle’. There are also rest groups to classify unknown particles based on similar morphology for possible future identification. While some of these non-biological and’unknown’ categorizations hold little research value, we label them to improve training of machine learning algorithms.

The previously published FlowCam biodiversity assessment archives in EurOBIS, OBIS, MDA and the LifeWatch Data Explorer have been fully updated and now include these most recent taxonomic reviews of the full time series. All currently recognized groups with and AphiaIDs from the World Register of Marine Species taxonomic backbone (WoRMS)^38^ are listed in Supplementary table 1. This new refined taxonomic structure of the dataset not only increases the accuracy of biodiversity occurrence data but also results in more homogenous groups leading to increased accuracy of model predictions. CNNs were retrained on the taxonomically revised dataset and can now recognise 95 groups as opposed to 53 groups in 2019. Despite the time-consuming nature of these revisions, regular review of data guarantees high quality throughout the time series.

## Data Records

The biodiversity occurrence and density data are aggregated to taxon counts and densities per sample post-validation on a month basis and made available under a CC-BY 4.0 license. All dataset records are summarized in Table 2, these datasets will be updated annually with new data releases.

**Table 2:** Data records of processed FlowCam datasets published in public repositories.

dataset	format	repository	License	Description
Biodiversity data	DwC-A	EurOBIS, OBIS^40^	CC-BY 4.0	Aggregated biodiversity data, taxon densities and counts per sample
Annotated image library	zip	MDA, Zenodo^43^	CC-BY 4.0	Full annotated image library, encompassing every particle images and its label for all samples May 2017-August 2024
training dataset	zip	Zenodo^44^	CC-BY 4.0	Training data split used in CNN training

The biodiversity occurrence and density are annually archived in European and global open-access repositories like the European and global node of the Ocean Biogeographic Information System (EurOBIS and OBIS). These archives require the Darwin Core Archive (DwC-A) format as this is considered the most suitable format for sample-based biodiversity data. Sampling time and spatial information are stored in a single “Event Core” text file while the “Occurrence Core” holds the occurrence data, where all taxon names are matched against the WoRMS taxonomic backbone^38^. Sampling descriptions and measured values are stored in the “Extended MeasurementOrFactExtension” or “eMoF” text file. Associated parameters on water quality, and essentially temperature, salinity, are included in the extension. All data within this format are linked to domain-specific controlled vocabularies developed by the British Oceanographic Datacentre^39^, which are accessible web services (P01 for identifying marine environmental and biological measurements, P06 to identify units, and L22 to define sensors and instruments). The FlowCam dataset is available in OBIS via 10.14284/710 and EurOBIS via 10.14284/650^40^.

The obtained DwC-A is further archived in the Marine Data Archive (https://marinedataarchive.org/) as well, an online repository specifically developed to independently archive data files in a fully documented manner. Subsequently, the archive is assigned a digital object identifier (DOI) for traceability. The data in MDA is available via 10.14284/710^41^.

All datasets described above are linked in the Integrated Marine Information System (IMIS), an ISO-19115 compliant catalogue for metadata discovery catalogue. The parent record of the FlowCam biodiversity assessment dataset in the BPNS is available via 10.14284/650^42^.

The full annotated image library, i.e. the ROIs and their quality controlled labels, is published in the Marine Data Archive and available via 10.14284/680 as well as Zenodo via 10.14284/680^43^. The training dataset sampled from this image library and used in our latest CNN training is available through Zenodo via 10.5281/zenodo.10554845^44^.

## Technical Validation

FlowCam is a trusted technology with hundreds of peer reviewed publications on applications of the technology in the fields of particle analysis in biopharma, environmental monitoring, water-quality assessment, aquaculture and quality control programs across a wide range of manufacturing industries. Since its first presentation in Sieracki *et al*., 1998 as an instrument for automated analysis of marine microplankton, the technology has been widely applied in plankton monitoring, with a couple of dozen instruments in Europe alone routinely used for environmental monitoring.

All images are labelled in a controlled way to assure high accuracy and continuity in the time series. The inference of the images in a first classification step speeds up manual validation in a first step, but all predicted images are manually checked and corrected where necessary by scientists familiarized with phytoplankton taxa in the BPNS. The recent taxonomic review of the dataset has led to a significant increase of taxonomic resolution, with all previously published data revisited. The changes in labelling rules were mutually agreed upon with the previous author Luz Amadei Martínez, and are based on a large body of identification and reference taxonomic literature provided by the Advanced Phytoplankton Course (APC12), and initiative supported by supported by UNESCO and its Intergovernmental Oceanographic Commission (IOC), the IOC Science and Communication Centre on Harmful Algae (IOC UNESCO / SCCHA) and Ocean Teacher Global Academy (IOC UNESCO / OTGA).

Bio archives of the dataset via EurOBIS, OBIS, EMODnet and the LifeWatch Data Explorer undergo regular and metadata checks and curation and adhere to international standards, vocabularies and the WoRMS taxonomic backbone.

## Usage Notes

It is important to note that for certain bloom taxa, like *Rhizosolenia* (T. Brightwell, 1858) and *Bellerochea* (H. Van Heurck, 1885), clogging issues can introduce biases in density estimates and caution is advised to dataset users in dealing with heavy bloom samples. The annually recurrent *Rhizosolenia* summer bloom poses significant challenges during laboratory processing due to severe clogging issues, resulting in biased density estimates. These bloom events are characterised by exceptionally high loads of long, elongated cells that surpass the prefilter in the apical axis direction and subsequently clog flow cells as they turn in the pervalvar axis direction. These cells have the tendency to adhere to other cells and flow cell walls by their processes, resulting in clogging of flow cells and inflation of PPUI (Particles Per Use Image) measurements in presample runs. These elevated PPUI estimates lead to extreme dilution factors and subsequent high density estimates (see Eq. 1). As the dilution factor of a sample is used in the denominator for the density calculation of each taxon in the sample, this overestimates the density of not only the bloom taxon itself, but all other taxa present in the sample, and this effect is not visible in cell counts or relative abundances. *Rhizosolenia* and long (chained) diatoms, often used as a proxy for broken *Rhizosolenia* cells when the ends have broken off, will be dominant in samples that show extreme high outlier densities in comparison with other sample densities. A similar issue of clogging of flow cells inflating cell densities was observed during an extreme *Bellerochea* bloom event in 2022. This is the result of a technical limitation of the technology and method we use. We advise dataset users to be mindful of these taxa in bloom samples during analysis on untransformed density values as FlowCam may inaccurately handle these taxa during bloom events, resulting in biased density measurements for all taxa in the bloom sample. We advise to work on abundances data instead of densities, as this is not impacted by the dilution factor or discard extreme outliers when working with density data.

A second limitation to consider is the bias introduced by the width of the flow cell and matched pre filter of 300 µm. Taxa that exceed the 300 µm prefilter in terms of cell or colony dimensions will be biased in terms of abundance and/or densities, and for these taxa only the proportion of cells or broken colonies will pass the prefilter and be imaged. A good example of this is *Noctiluca* (Suriray, 1836), which is almost exclusively seen as ruptured cells and remnants in images, and are therefore placed under the ‘Noctilucales’ label instead of the ‘*Noctiluca scintillans*’ label, as torn cells don’t always show the necessary morphological characteristics for species or genus level identification. While the presence of *Noctiluca* can be detected in the dataset, cell counts and densities are not accurate cell counts. Another example is *Phaeocystis*, observed annually during a spring bloom but difficult to accurately estimate in terms of abundance or densities using FlowCam as the 300 µm pre filter removes the majority of the colonies. While we can detect the presence of *Phaeocystis* blooms in images, the colonies often appear ruptured and severely degraded, with sometimes only the gelatinous matrix present. Consequently, these images are labelled under ‘Phytoplankton Colony’. Other taxa that have an ESD larger or equal to 300 µm are Zooplankton taxa (Appendicularia, Copepoda adult, Crustacea, Crustaceae:part, Decapoda, *Litonotus*, Nauplii, Nematoda, Ophiuroidea/Echinoidea larvae, Polychaeta, Zooplankton), some large and/or colonial diatoms (*Bacillaria paxillifer, Bellerochea, Chaetoceros, Guinardia striata/Dactyliosolen phuketensis*, Long (chained) diatom, *Plagiogrammopsis/Bellerochea malleus, Proboscia indica, Pseudo-nitzschia, Rhizosolenia, Rhizosolenia setigera (f. pungens)/R. hebetata f. semispina, Stephanopyxis*, *Thalassiosira/Porosira, Trieres sinensis*) and dinoflagellate *Tripos fusus*. Caution is advised when using abundance or count data for these taxa.

## Supplementary information

Supplementary table 1

## Data Availability

The FlowCam biodiversity occurrence and density data are available in the global and Euopean node of the Ocean Biogeographic Information System (OBIS and EurOBIS) and in the Marine Data Archive (MDA). The data in these archives is stored in the Darwin Core Archive (DwC-A) format for sample-based biodiversity data. Sampling time and spatial information are stored in a single “Event Core” text file while the “Occurrence Core” holds the occurrence data, with all taxon names matched to the WoRMS taxonomic backbone^38^. Sampling descriptions and measured values are stored in the “Extended MeasurementOrFactExtension” or “eMoF” text file. Associated parameters on water quality, and essentially temperature, salinity, are also included in this extension file. The FlowCam dataset is available in OBIS via 10.14284/760 and EurOBIS via 10.14284/760^40^ and in the Marine Data Archive via 10.14284/710^41^. The full annotated image library, i.e. the ROIs and their quality controlled labels, is published in the Marine Data Archive and available via 10.14284/680 as well as Zenodo via 10.14284/680^43^. The training dataset sampled from this image library and used in our latest CNN training is available through Zenodo via 10.5281/zenodo.10554845^44^. All datasets described above are linked to each other in the Integrated Marine Information System (IMIS) catalogue for metadata discovery catalogue via 10.14284/650^42^.

## Code availability

Code to process raw FlowCam data is not openly accessible as it is fully tailored to and dependent on VLIZ internal data systems (BioSense MongoDB, MIDAS RV information system), the LifeWatch monitoring framework and methodological which makes it not applicable for other FlowCam users. It is a means of processing large amounts of raw data and linking to VLIZ internal data systems, but is in no way imperative for processing FlowCam data. A very similar flow can easily be archived via the EcoTaxa platform for instance (Picheral M. *et al*., https://ecotaxa.obs-vlfr.fr/). The internal pipeline was developed at a time where the EcoTaxa platform was not suitable yet for processing and classifying FlowCam data. As part of the Imaging data and services for aquatic science (iMagine) Horizon 2020 project, we are committed to publishing the full FlowCam human annotated image library comprising over 2 million images, training data splits and trained CNN. Currently, a training dataset is openly available via Zenodo^44^ and the trained CNN is available for external use via the iMagine platform at https://dashboard.cloud.imagine-ai.eu/marketplace/modules/uc-lifewatch-deep-oc-phyto-plankton-classification. Source code of the classification service is publicly available via https://github.com/lifewatch/phyto-plankton-classification under an Apache 2.0 license. As the project is still ongoing, no static identifiers can be assigned to the model code yet. By October 2025, a DOI will be assigned and code will be static.
